# Observation of practical teaching skills instrument (OPTIn): transforming the way we observe, evaluate and improve physically active teaching strategies

**DOI:** 10.3389/fspor.2026.1748938

**Published:** 2026-02-17

**Authors:** Natalie Lander, Kira Patterson, Narelle Eather, Katie Robinson, Nick Riley, Samuel K Lai, Ned Weatherell, Jess Orr, Jo Salmon

**Affiliations:** 1 Institute for Physical Activity and Nutrition (IPAN), School of Exercise and Nutrition Sciences, Deakin University, Geelong, VIC, Australia; 2 School of Education, College of Arts, Law and Education, University of Tasmania, Hobart, TAS, Australia; 3 Centre for Active Living and Learning, College of Human and Social Futures, University of Newcastle, Callaghan, NSW, Australia

**Keywords:** evaluation, measurement, pedagogy, physical activity, reflection

## Abstract

**Background:**

Teaching effectiveness is a key determinant of student learning outcomes. Increasing evidence supports the use of physically active teaching strategies to not only enhance student learning engagement and achievement, but also health. However, existing classroom teaching observation tools often fail to assess these active pedagogical approaches, particularly among generalist teachers. This study details the development and evaluation of the *Observation of Practical Teaching Skills Instrument* (OPTIn), designed to assess both general teaching effectiveness and the implementation of physically active strategies.

**Methods:**

OPTIn was developed through a four-phase process. Initially, key domains were identified via expert consensus (*n* = 6), then refined with feedback from education and physical activity specialists (*n* = 9). The instrument was piloted in authentic classroom settings by teacher educators (*n* = 5). Reliability was then examined through inter- and intra-rater agreement analyses with three independent raters across five lesson observations. Content validity was assessed by comparison with expert evaluations.

**Results:**

The final instrument includes seven domains aligned with national teaching standards, evidence-based practice and physically active teaching. Feedback confirmed the tool's clarity, relevance, and feasibility, with recommendations to expand indicators for active teaching and incorporate peer/self-assessment features. Inter-rater reliability ranged from 66.7% to 94.7%, while intra-rater agreement showed minimal variation (93.3% and 86.7%). High agreement with expert ratings supported content validity.

**Conclusion:**

OPTIn provides a novel, practice-informed tool for observing and improving active teaching strategies. Its use can support teacher professional development and contribute to advancing the measurement of physical activity integration within educational practice.

## 1 Introduction

The effectiveness of teaching has a profound impact on student outcomes, with numerous studies affirming the correlation between teacher quality and student achievement (1–6). High-quality teaching, characterised by strong instructional clarity, effective classroom management, meaningful feedback, high expectations, and adaptive use of evidence-based pedagogical strategies is consistently associated with higher student achievement, with effects observed across standardised test performance, learning gains over time, and longer-term outcomes such as educational attainment (1–6). Evidence-based pedagogical strategies often underscore the significance of three primary dimensions of teacher effectiveness: teacher experience, knowledge, and behaviour (7–10). Each of these dimensions has shown to influence student learning, with teacher behaviours, such as instructional approaches and content delivery (pedagogical content knowledge), playing a particularly pivotal role (11). When teachers employ evidence-based methods and instructional practices, student outcomes improve significantly (3, 5).

A growing body of literature has explored the integration of physical activity as an effective instructional approach, highlighting its positive influence on cognitive and academic outcomes (12–16). Physically active teaching strategies (e.g., embodied learning, experiential learning, active breaks) have been linked to enhanced attention, memory retention, and overall academic achievement (12, 15–17). Regular physical activity has also been associated with improvements in mental health, self-regulation, and social development, factors that further enhance learning (12, 13, 18). In addition, physically active instructional approaches facilitate several evidence-based pedagogical strategies, including collaborative opportunities for learning, differentiation, multiple exposures of content, and the use of metacognitive strategies (19). These pedagogical strategies are recognized to positively impact learning (20) and have been consistently linked to improved student outcomes across various educational settings (12, 21). While the benefits of physically active instructional approaches are well-documented (22), there remains a gap in understanding how teacher actions influence the successful implementation of such strategies, and subsequently the impacts of these strategies on student outcomes.

Observation, feedback, and reflection are crucial for evaluating and enhancing teaching effectiveness (23, 24). Together, they create a cycle of continuous professional growth, enabling teachers to critically evaluate and improve their teaching practices through evidence and self-awareness, refine instructional methods, and better address student needs (8, 25–28). Several instruments have been developed to observe, assess and provide feedback on teacher practice in the classroom (29, 30). These tools are often used for research, professional development, or teacher evaluations. Some notable examples of instruments for classroom use include: (1) the Classroom Assessment Scoring System (CLASS) (31), which focuses on emotional support, classroom organization, and instructional support; (2) the Framework for Teaching (FFT) (32), which is widely used for teacher evaluations and professional development and outlines aspects of teaching that contribute to student learning across four domains—planning and preparation, classroom environment, instruction, professional responsibilities; and (3) the Reformed Teaching Observation Protocol (RTOP) (33), where observers assess how much the teaching encourages inquiry, problem-solving, and student engagement.

In Australia, the Quality Teaching Model (34) was developed specifically for the state of New South Wales, as part of a focus on improving the quality of teaching and learning by assessing teaching based on three dimensions: intellectual quality, quality learning environment, and significance**.** The model was developed as a self-reflection tool to be used by teachers to understand, analyze and focus their own teaching practices for improved student learning. In South Australia, the Teaching for Effective Learning (TfEL) Observation Tool was developed by the South Australian Department for Education (35). It is aligned with Australian educational standards and focuses on building teachers' professional practice in areas such as personalizing learning, engaging learners, and creating safe learning environments. These instruments are usually used by trained observers who apply rubrics or specific criteria to score the effectiveness of teaching practices. Each tool has its specific focus depending on the subject matter, grade level, or pedagogical framework. These observation tools focus on traditional teaching methods, and despite its importance, do not account for the integration of physical activity in teaching practice.

Several observation tools, such as System for Observing Fitness Instruction Time (SOFIT) (36) and the Observational System for Recording Physical Activity in Children (OSRAC) (37) have been developed to assess student-level physical activity in physical education contexts. While these instruments provide valuable insights into student behaviours and lesson context, they offer limited information on the specific teaching practices driving those behaviours. More recently instruments have been developed to assess key instructional competencies in Physical Education (PE), such as System for Observing Teaching Competencies in Physical Education (SOTC-PE) (38). This tool systematically evaluates whether teachers are delivering high-quality PE lessons by capturing observable teaching behaviours. While tools like the SOTC-PE provide valuable insight into teaching quality within physical education lessons, their focus is limited to a subject that is typically delivered once per week. In contrast, children spend the majority of their school day with their generalist or classroom teacher, in environments where sedentary behaviour is both pervasive and often reinforced by traditional pedagogical practices. Consequently, there is a critical need for observational tools that capture instructional behaviours in generalist settings, particularly strategies that embed physically active learning. Without assessing teaching practices across the broader school day, it becomes difficult to understand the full scope of influences on student behaviour, engagement, and health. Observing and measuring classroom teacher effectiveness in this context is therefore essential to designing meaningful, scalable interventions that address inactivity and support positive behavioural and academic outcomes.

Teacher practice is central to student outcomes, particularly in creating environments that support physically active learning (17, 39). Despite the benefits of active teaching for engagement, health, and learning, no validated instruments exist to objectively observe these strategies in classrooms. This gap limits our understanding of how teacher behaviours shape outcomes. Developing such a tool will enable systematic evaluation of active teaching practices and their impact on student physical activity, health, and learning. The aim of this study was to develop an instrument for evaluating classroom teachers' practical teaching skills, with a focus on physically active instructional approaches, namely the Observation of Practical Teaching Skills Instrument (OPTIn). To ensure its applicability, the aim was to develop a tool to be adaptable, and therefore appropriate for the evaluation of different levels of teaching proficiency (e.g., graduate, proficient, expert) of generalist (classroom) teachers in primary, secondary and tertiary sectors.

## 2 Methods

The development of OPTIn was guided by a hybrid theoretical framework combining Shulman's (11) Pedagogical Content Knowledge, Constructivist Learning Theory (40), and evidence-based models of teaching effectiveness (5, 27). These frameworks collectively underscore the importance of teacher knowledge, behaviours, and instructional strategies in promoting student engagement and achievement. Additionally, the Ecological Model of Physical Activity (41) informed the integration of physical activity within educational contexts, recognizing the role of teacher behaviours in shaping active learning environments. This theoretical alignment ensured OPTIn captured the multifaceted nature of teaching effectiveness in physically active classrooms and supported its application across diverse educational contexts.

The tool development included four distinct phases. In the first phase, the lead researcher assembled an expert working group to guide the instrument's development. During the second phase, feedback was solicited from 10 additional experts to evaluate whether the instrument accurately measured its intended constructs. The instrument was subsequently refined based on the expert input, and a comprehensive training guide was developed. In the third phase, a pilot test was conducted with a small sample of experts, not previously involved in instrument development, to assess the instrument's practical application. Detailed feedback was gathered, which contributed to the final refinements of the instrument. In the final phase, the reliability and validity of OPTIn were evaluated by comparing scores from five lessons, assessed independently by two trained raters and a content expert. Each phase is detailed below. Ethics approval for the study was granted by Deakin University (HAE-20-170).

### 2.1 Phase 1: development of an expert working group

The lead researcher sent email invitations to academics and/or teacher educators who had previously participated in the implementation of the TransformUs Higher Ed program, an initial teacher education (ITE) initiative aimed at equipping future teachers with innovative pedagogical, behavioural, and environmental strategies to promote movement-based learning opportunities for primary school students (42–44). Six researchers and/or teacher educators from five Australian universities were invited to join a working group; all accepted the invitation and collaborated with the lead researcher. The working group comprised of experts in relevant domains, such as teaching and learning, teacher education, and physical activity. This expertise ensured that the collective set of items developed for OPTIn adequately captured the breadth of the construct. The expert working group conducted a thorough examination of relevant literature, government policies, teaching standards, and professional recommendations to gather evidence for the development of the initial items for OPTIn.

### 2.2 Phase 2: expert judgement

Once the initial items were developed, the first version of OPTIn was compiled in Qualtrics (45), and a link was distributed to a group of external expert reviewers with expertise in the relevant content area. The expert review provided a fresh set of eyes to critically look at the items and has become common practice in instrument development (46–48). The panel of experts included researchers in education and physical activity, as well as teacher educators and practicing teachers. Researchers were identified via Expertscape, teacher educators were located through university websites, and practicing teachers were recruited through working group networks. Invitations were sent to 25 potential experts, of which 10 accepted and provided written informed consent to participate. This number is consistent with the recommended range for expert reviewers in the literature (47). Experts included physical activity researchers (*n* = 2), teachers (*n* = 2), education researcher (*n* = 1), Education course director (*n* = 1), teacher educator/academic (*n* = 3). These expert reviewers were distinct from the working group that developed the original pool of items, ensuring an independent, unbiased perspective.

The purpose of the expert review process was to pre-test the instrument and enhance its content validity by gathering feedback on the relevance of each item in relation to the construct being measured (49). The pre-testing focused on evaluating the content, format, wording, and layout of OPTIn, while identifying and eliminating any sources of confusion, ambiguity, or bias. This process also contributed to improving the relevance, clarity, and readability of the instrument (50).

Expert opinions were solicited in two ways. First, using a 4-point Likert scale (i.e., strongly disagree, disagree, agree, strongly agree), expert reviewers were asked to rate the extent to which each survey item represented the content area of the proposed construct. Open-response options accompanied the Likert scale questions, allowing reviewers to provide explanations for their ratings. Key questions included:

1. Does the instrument appear to measure what it is intended to measure?
2. Are the items clear and understandable?
3. Do the items cover all relevant and important aspects of the concept?
4. Are there any elements irrelevant to the concept?
5. Is the content of the instrument appropriate for the target population?

Second, expert reviewers were invited to provide additional comments on any aspect of the instrument using a Microsoft Word document or via a recorded and automatically transcribed Zoom call, offering further feedback to refine OPTIn. Feedback from the experts were used to develop a second and refined version of the tool, and a comprehensive user guide.

### 2.3 Phase 3: pilot testing

Five expert reviewers, including four education academics and one professional practice coordinator/placement supervisor, were invited to pilot test the second version of OPTIn, in real-world settings. Teacher educators and placement supervisors were located through university websites. Invitations were sent out to 15 participants, of which five accepted and provided written informed consent to participate. Each expert pilot tested OPTIn a minimum of three times, and this occurred during pre-service professional teacher placements in schools, pre-service micro-teaching sessions at universities, or in university seminars and tutorials.

The pilot test served two main purposes. First, it provided a small-scale trial of OPTIn's practical utility and implementation in the field. Here, the instrument was administered to a subset of the target population (e.g., pre-service teachers, teachers, teacher educators), aligning with the recommended sample size for pilot studies (typically 10 to 30 participants). Data were collected and analyzed using the same observation techniques and Qualtrics software planned for real world use. The results were evaluated, and any issues with the instrument or methodology were addressed and modified accordingly.

Second, the five expert reviewers provided written feedback to share their experiences using the instrument in practice. Initially, teacher educators were asked to recall their experiences using OPTIn without specific prompts. They were then guided through a series of structured questions about the overall tool, including: (a) Did you understand how to use the instrument in class/seminar? (Please explain.); (b) Was the flow of the questions easy to follow?; (c) Did the responses adequately reflect your understanding of the questions?; (d) Did the instrument omit anything you considered important?; and (e) Do you have any additional comments?

All documents were reviewed, with any necessary revisions made, particularly where teacher educators raised concerns or highlighted issues with the instrument's use.

### 2.4 Phase 4: inter- and intra-rater reliability and content validity of OPTIn

To assess the inter- and intra-rater reliability and content validity of the revised version of OPTIn, two sessional teacher educators were invited via email, provided written consent, and were recruited as raters. Each rater received the detailed user guide (Supplementary File) outlining the instrument's purpose, structure, and behavioural criteria. A two-hour online training session, conducted by the lead researcher, familiarised raters with the tool's dimensions, allowed practice scoring a sample video lesson, and included discussion of key terms to ensure consistent interpretation and reduce observer drift.

Following training, the raters independently viewed and scored five pre-recorded lessons of pre-service teachers delivering a 45 min primary school lesson during their placement, and used OPTIn to assess teaching in each lesson. Raters systematically documented the presence of each physically active teaching strategy observed during the lessons. For every occurrence, selections were made using dropdown menus in Qualtrics to identify: (i) the specific strategy (e.g., active environment, active break, active academic lesson); (ii) the type of strategy, such as signage or equipment for active environment, energizers or transitions for active breaks, and experiential or incidental approaches for active lessons; (iii) the phase of the lesson in which the strategy occurred (introduction, body, conclusion); (iv) associated evidence-based teaching practices (e.g., goal setting, feedback, collaborative learning, metacognitive strategies); and (v) the relevant teaching standard addressed (e.g., knowing students and how they learn, knowing content and how to teach it, planning and implementing effective teaching, and creating supportive learning environments). This detailed categorization followed the OPTIn instrument's event-based structure, recording and defining each strategy occurrence, resulting in multiple strategies and types being identified per lesson.

To assess intra-rater reliability, lesson one was re-scored by each rater after a seven-day interval. Inter- and intra-rater reliability were calculated using percentage agreement per lesson, based on the number of behaviours where agreement was achieved (observed or not observed), divided by the total number of behaviours scored, multiplied by 100. Inter-rater agreement was averaged across all lessons to determine overall reliability.

To assess content validity, a content expert who was a member of the expert working group, also independently scored all five lessons using the OPTIn tool. The expert's ratings were treated as a criterion standard, against which the two trained raters' scores were compared. This comparison provided an estimate of validity, based on the extent to which rater scores aligned with those of the expert, supporting the accuracy and appropriateness of the OPTIn instrument for capturing intended teaching behaviours.

## 3 Results

### 3.1 Phase 1

The expert working group identified seven domains that formed the initial items for OPTIn. These included key aspects of active teaching, lesson planning, evidence-based teaching practices, teaching standards, teaching competence and confidence and student engagement. Table 1 provides an overview of each domain, with evidence and alignment (where relevant) to the Australian Professional Standards for Teachers (APST) (51). These standards provide a framework for understanding how strategies such as multiple exposures, differentiation, explicit teaching, feedback, planning, and metacognitive strategies contribute to high-quality teaching and improved student outcomes in the Australian context.

**Table 1:** Initial domains and items developed for the OPTIn.

Domain	Item	Elaboration	Evidence
Active Instructional Approaches	Creating an active classroom environment	The use of signage, equipment, facilities, resources, classroom layout/desk configuration and policy, to support or promote meaningful physical activity in the classroom	(Salmon et al., 2020)
	Integrating active breaks	The use of short active breaks to break prolonged sitting and complement lesson content. Active breaks can be used for physical and visual reinforcement, to introduce or summarise lesson content, to structure the lesson, to transition the lesson, to proactively manage the class, and to create a positive classroom environment.	(Salmon et al., 2020)
			[Lander et al., (19)]
	Delivering active academic lesson	Active lessons utilize incidental or structural activity, or embodied or experiential learning, to change the delivery of a traditional seated class lesson to one where the body or the movement becomes a vehicle for learning.	(Salmon et al., 2020)
			(Lander et al., (42); Lander et al., (43); Lander et al., (44)
Lesson Phase	Introduction	To capture students' attention, activate prior knowledge, and set the context for the lesson. It helps students understand the learning objectives and prepares them for new information.	(Hattie, (5)
			(Rosenshine, 2012)
			(Mayer, 2004)
			[Australian Institute for Teaching and School Leadership (AITSL), 2011]
			[Hollingsworth & Ybarra, (55)]
	Body	The body of the lesson involves the core content, where new concepts, skills, or knowledge are taught through various activities, explanations, and discussions.	[Hattie, (5)]
			[Australian Institute for Teaching and School Leadership (AITSL), 2011]
			[Hollingsworth & Ybarra, (55)]
	Conclusion	To summarize key points, reinforce learning, and assess understanding, allowing for reflection or clarification. A clear conclusion consolidates learning, helping students retain information and transfer it to future contexts, while also providing an opportunity for feedback.	[Hattie, (5)] (Rosenshine, 2012)
			[Australian Institute for Teaching and School Leadership (AITSL), 2011] [Hollingsworth & Ybarra, (55)]
Evidence-based teaching practices	Goal Setting	Goal setting is used to define clear, specific objectives that guide instructional strategies and student learning outcomes. It is a systematic process that enables teachers to articulate what they want students to achieve during a lesson or unit, ensuring that the teaching aligns with curriculum standards and student needs.	(Zimmerman & Kitsantas, 2014)
	Structuring Lessons	Structuring lessons refers to the deliberate organization and sequencing of instructional activities to promote optimal student engagement and learning.	[Australian Institute for Teaching and School Leadership (AITSL), 2011]
	Collaborative Learning	Providing opportunities for students to work together in small groups to achieve a common learning goal. This method emphasizes peer interaction, active participation, and the exchange of ideas, enabling students to engage deeply with the material through discussion, problem-solving, and shared responsibilities.	(Slavin, 2014)
			(Millis, 2023)
	Metacognitive Strategies	Metacognitive strategies, refer to the techniques used to help students become aware of and regulate their own thinking and learning processes, improve their ability to plan, monitor, and evaluate their understanding.	(Zohar & David, 2008)
	Explicit Teaching	Explicit teaching involves clear instructions, modelling, and scaffolded learning experiences to ensure that students understand and apply concepts effectively.	[Hattie & Timperley, (26)]
			(Rosenshine, 2012)
	Multiple exposures	Using repeated, varied exposures to content to help students consolidate learning over time, ensuring better retention and understanding.	[Australian Institute for Teaching and School Leadership (AITSL), 2011]
			(Brown, 2011)
	Differentiation	Differentiated teaching refers to the practice of tailoring instruction to meet the diverse needs, abilities, and learning styles of individual students within a classroom.	[Tomlinson, (21)]
	Questioning	Questioning engages students, promotes critical thinking, and deepens understanding. By using open-ended and well-timed questions, teachers encourage active participation, assess comprehension, and stimulates higher-order thinking and problem-solving skills.	[Black & Wiliam, (56)]
			(Chin, 2006)
	Worked examples	Worked examples provide step-by-step demonstrations of problem-solving processes, helping students understand complex tasks. By observing correct methods and rationales, students can focus on learning key principles before attempting independent problem-solving.	(Sweller et al., 2011)
			(Renkl, 2014)
	Feedback	Providing timely, constructive feedback enables students to understand their progress and areas for improvement, enhancing learning outcomes.	[Hattie & Timperley, (26)]
			[Australian Institute for Teaching and School Leadership (AITSL), 2011]
Teaching Standard	Know students and how they learn	Understand the diverse needs, backgrounds, and learning styles of students to adapt teaching strategies effectively.	[Australian Institute for Teaching and School Leadership (AITSL), 2011]
	Know the content and how to teach it	Master the curriculum content and employ appropriate teaching methods for student understanding.	[Australian Institute for Teaching and School Leadership (AITSL), 2011]
	Plan for and implement effective teaching	Design well-structured lessons and implement teaching strategies that engage students and promote learning.	[Australian Institute for Teaching and School Leadership (AITSL), 2011]
	Create and maintain supportive and safe learning environments	Foster a positive, inclusive, and safe classroom environment that encourages participation and student well-being.	[Australian Institute for Teaching and School Leadership (AITSL), 2011]
	Assess, provide feedback and report on student learning	Use various assessment methods to monitor progress, provide constructive feedback, and report results clearly to students and stakeholders.	[Australian Institute for Teaching and School Leadership (AITSL), 2011]
Teacher characteristics	Teacher confidence	Teacher confidence, the belief in one's ability to teach effectively, enhances classroom management, instruction, and student engagement. Confident teachers are more likely to try new strategies, foster positive environments, and improve student outcomes and professional growth.	(Tschannen-Moran & Hoy, 2001)
			(Klassen & Tze, 2014)
	Teacher competence	Teacher competence refers to the knowledge, skills, and professional attitudes required for effective teaching. It includes mastery of subject content (content knowledge), pedagogical expertise (pedagogical content knowledge), classroom management, and the ability to assess and support diverse learners. High teacher competence is essential for student achievement, engagement, and the overall quality of education.	(Shulman, 2011) (Darling-Hammond, 2021)
			(Darling-Hammond, 2021)
Student Characteristics	Student engagement	Student engagement refers to the degree of attention, curiosity, and interest students show in learning. It is key to academic success and includes behavioural, emotional, and cognitive involvement. Effective teacher practices to boost engagement include active learning, relevant content, interactive discussions, timely feedback, and fostering a supportive classroom environment.	(Fredricks et al., 2004)
			(Reeve, 2012)

**Figure 1:**
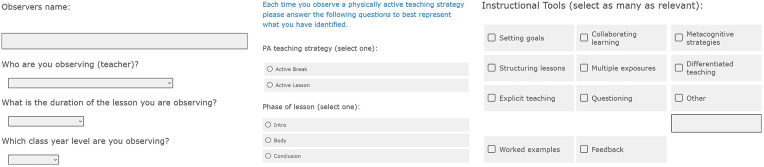
Landing page, Observer and observee details, Active Strategy, Lesson Phase, Instructional Tool and Teaching Standard.

The initial version of OPTIn was developed on the Qualtrics platform (45), incorporating key elements identified by the working group. The tool included a landing page outlining the rationale and brief instructions, followed by sections capturing details of the observer and observee, the active strategy employed, the lesson phase, instructional tools used, and the relevant teaching standards (see Figure 1 for examples).

### 3.2 Phase 2

Nine of the 10 experts returned their ratings and reported the extent to which the initial version of the OPTIn tool represented the content area of the proposed construct. The results of their ratings against the five questions are presented in Table 2.

**Table 2:** Expert judgement rating.

Items	Strongly agree	Agree	Disagree	Strongly disagree
	%	%	%	%
Appears to measure what it intends to measure	62.5	37.5		
Items clear and understandable	50.0	50.0		
Items cover all relevant aspects of the concept	62.5	37.5		
Any irrelevant elements				100
Content appropriate for target audience	100			

**Figure 2:**
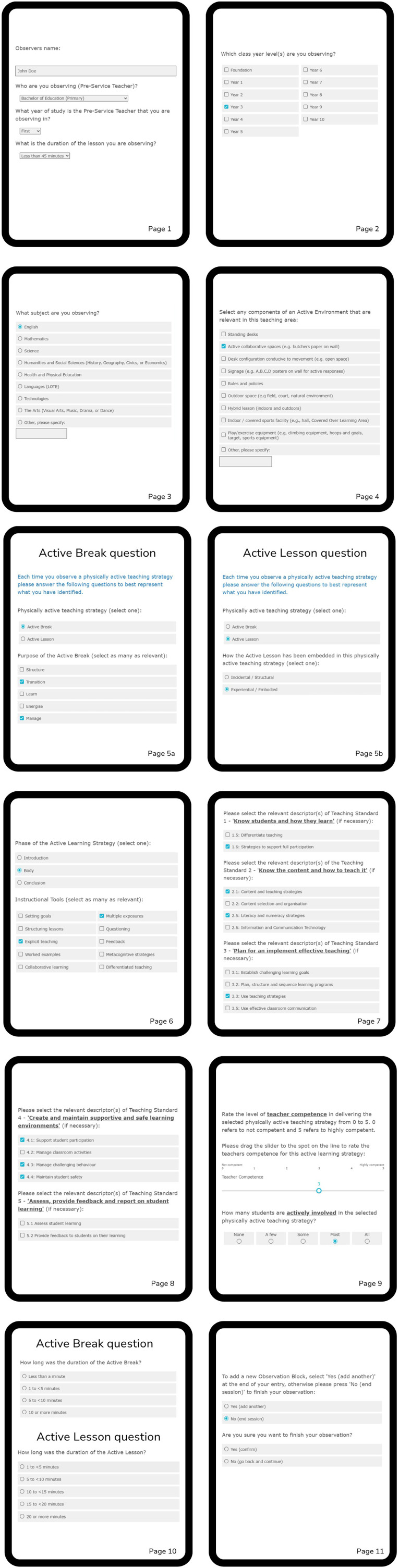
Who is being observed example questions, Components of observed active environments, Observed physically active teaching strategy.

Comments and recommendations were also provided by the experts on any aspect of the instrument. Key recommendations were around the structure, flow and level of detail as summarized below:

- Reduce the length of introductory text or incorporate a skip function to allow users to proceed directly to the observation phase.
- Add more detail regarding observe, allow for pre-service teacher observation (See Figures 2a–c for example).
- Separate the questions regarding class/lesson and year/grade level from those related to the subject being taught.
- Broaden the range of year/grade level options (See Figure 2 for example).
- Provide concrete examples of active environments (e.g., signage, classroom layout, resources) for observers to identify during the observation (See Figure 2 for example).
- Offer examples for categorizing different types of active breaks observed (See Figure 2 for example).
- Include clear definitions of key concepts such as “embodied learning” to ensure consistent understanding.
- Make the subsections or descriptors of teaching standards readily visible to aid observer recall.
- Consider measuring both the duration of student activity and the number of students actively engaged during lessons.
- Reevaluate the feasibility of assessing teacher confidence through observation; instead, focus on observing teacher competence.
- Ensure consistency in terminology throughout the instrument (e.g., use of “teacher strategy” vs. “teacher practice”).
- Add a final confirmation page before submission to allow for review.
- Include a “back” function to enable corrections during data entry.
- Incorporate a section for observer notes to capture additional insights.
- Provide a glossary or user manual to support users in understanding terms and processes.

Expert judgment feedback was incorporated into version two of OPTIn. Examples are presented in Figures 2a–c below.

### 3.3 Phase 3

Version two of the OPTIn tool was pilot-tested in the field by experts. Each expert used the tool to observe pre-service teachers during their professional practice placements. Based on the written feedback from the experts, four key points emerged regarding the practical utility and implementation of the OPTIn tool in the field:

1. The tool was perceived as easy to follow, with a logical flow.
2. It effectively captured key aspects relevant to beginner teachers and effective teaching.
3. Experts recommended integrating a built-in user guide rather than relying on a separate manual.
4. There were suggestions to include an option for peer assessment or self-assessment via video, facilitating PST peer feedback and self-reflection.

#### 3.3.1 Expert reflections

When prompted to reflect on the tool without specific guidance, the experts generally provided positive feedback regarding its practical use following the pilot testing. For example, one participant stated:

“I think the tool generally works well and could be a valuable addition to practicums.” (Professional placement coordinator)

The professional practice coordinator also shared:

“This will really add value to the way we evaluate teacher readiness. It could be used to demonstrate evidence against the teaching standards, which is the major assessment in their final year of the teaching degree”

Another participant noted:

“This will add a lot regarding the pre-service teacher's development. I could see it being used as part of regular observations but also as a self-assessment tool.” (Education academic 3)

#### 3.3.2 Structured responses to questions

All experts were able to understand the use of OPTIn in a classroom or seminar setting. They all agreed that the user guide was valuable in preparing them to use the tool, although two experts (Professional practice coordinator and Education Academic 4) suggested that an in-app guide might be more feasible than a separate user guide. Additionally, three experts (Education Academic 1, 2 and 3) noted that the tool's structure was well-aligned with the natural progression of a lesson, which facilitated a logical flow and clear focus on the instructional approach.

One of the experts suggested some elements of the tool were easier to use than others, with particular ease noted for the active break section due primarily to the added complexity and diversity in delivering active lessons “*I found it easier to apply for active breaks compared to active lessons. Active lessons often include many elements, making it challenging to treat them as a cohesive unit.*” (Education academic 2).

Experts consistently praised the logical flow and functionality of the questions and their response options. Experts highlighted the value of the dropdown functionality, which provided detailed elaborations for each selection. One expert remarked:

“Once the selection of instructional approaches was made, there were clear examples under each to enhance the specificity of the selection—such as the type of active break used.” (Education academic 4)

All experts agreed that the response options adequately reflected their understanding of the questions. Experts also emphasized that the depth of the response options allowed for greater specificity in the evaluation process. One expert stated:

“I like the way the teaching standards were explained with their elaborations or descriptors, allowing for a clear connection between the teaching strategy and the standard. This provides clear evidence of their practical teaching skills, which is what they are assessed on.” (Education academic 3)

This was supported by Expert 2 who said *“There were ample descriptors that helped provide detailed information on the lesson”.*

#### 3.3.3 Suggestions for improvement

One expert suggested that additional options for grade levels would be useful, as the current version of the tool did not accommodate composite or combined grades:

“Can we have an option for other grade levels, e.g., I observed a mixed 2/3 class?” (Education academic 1)

This expert also recommended expanding the tool's options for the type and intensity of physical activity, suggesting “*the inclusion of categories such as walking, running, high intensity activity, jumping etc would be helpful*” This suggestion was supported by Expert 2 who suggested that ‘*adding names for each activity would enhance reflection and feedback*’.

Expert 2 also recommended the expansion to the tool regarding strategy categorization, to provide more detailed and reflective assessment.

“I think it would benefit from additional clarification on specific sections, such as how teaching strategies are categorized (e.g., Incidental/Structural vs. Experiential/Embodied).”

#### 3.3.4 Omissions and additional considerations

When asked whether any important aspects had been omitted from the tool, the experts expressed general satisfaction with the key teaching aspects that were captured. One expert commented:

“The instrument enables great depth and detail, aligned with the key teaching standards relevant for graduate-level teachers.” (Education academic 3)

Another expert noted:

“The level of detail will enhance the feedback cycle as it provides explicit and specific areas where teachers are excelling and areas that may need improvement.” (Professional practice coordinator)

A few experts provided suggestions for further refinement, particularly related to the categorization of physical activity. One expert remarked:

“I was unsure how to include ’standing behind desks.’ I assume it refers to desk configuration, but it would be helpful to have an option for the type of activity.” (Education academic 1)

#### 3.3.5 Additional expert recommendations

The experts also provided valuable feedback regarding the use of an overarching scale for both the level of activity across the lesson and the level of teaching competency, according to the teaching standards. One expert recommended:

“I also think it would benefit PSTs if we could rate them against the standards on a 5-point scale. This is likely more in line with the observations they are measured against during practicum, which would be valuable for feedback and discussion.” (Education academic 1)

This was echoed by Expert 2 suggesting that “*a more detailed way to assess teacher competence and student engagement could enrich the tool's usefulness*”.

The same expert noted that added personalisation of each entry could be beneficial particularly at the pre-service level for specific and tailored feedback and self-reflection: “*Including options for student [pre-service teacher] names could make feedback more tailored*”.

### 3.4 Phase 4

The results of the inter- and intra-rater reliability and content validity analysis are summarised in Table 3. Overall, there was substantial agreement between the trained raters and the expert rater (gold standard) in identifying and coding physically active teaching strategies across the five video lessons.

**Table 3:** Inter- and intra-rater agreement between raters using the OPTIn.

Lesson	Rater	Strategies Identified	Matches with Gold Standard (R1)	Agreement with R1 (%)	Test–Retest Agreement
Lesson 1	Rater 1 (Gold Standard)	30	–	–	100% (30/30 same)
	Rater 2	26	24	80.0%	93.3% (28/30 same)
	Rater 3	24	24	80.0%	86.7% (26/30 same)
Lesson 2	Rater 1	20	–	–	–
	Rater 2	22	16	80.0%	–
	Rater 3	18	16	80.0%	–
Lesson 3	Rater 1	24	–	–	–
	Rater 2	22	20	83.3%	–
	Rater 3	20	18	75.0%	–
Lesson 4	Rater 1	18	–	–	–
	Rater 2	16	12	66.7%	–
	Rater 3	19	18	94.7%	–
Lesson 5	Rater 1	20	–	–	–
	Rater 2	18	16	80.0%	–
	Rater 3	16	14	70.0%	–

• Agreement scores calculated as: (Number of matching strategies with Rater 1 ÷ Number of strategies identified by Rater 1)   ×   100.

• Rater 1 served as both the gold standard and the content expert.

• Intra-rater reliability (test–retest) was assessed using repeated scoring of Lesson 1 after a 7-day interval.

• Raters were trained using a standardised guide and video-based calibration session.

Inter-rater reliability was generally high, with agreement ranging from 66.7% to 94.7% across lessons. The highest agreement occurred in Lesson 1, where both trained raters identified the majority of the strategies also captured by the expert rater. Agreement was slightly lower in Lesson 4 for Rater 2, suggesting potential variability in interpretation depending on lesson complexity or strategy visibility.

Intra-rater reliability, assessed by re-scoring Lesson 1 after a seven-day interval, demonstrated strong temporal consistency. Rater 1 (expert) identified the same 30 strategies upon re-assessment (100% agreement). Raters 2 and 3 showed only minor variation in the number of strategies identified at retest, with agreement levels of 93.3% and 86.7%, respectively. This suggests that once familiar with the OPTIn instrument, raters can consistently apply the criteria over time.

Content validity was supported through comparison with the expert rater's coding, which served as the criterion standard. The number of strategies identified by each trained rater that matched the expert's codes across lessons indicates the instrument's capacity to reliably capture intended teaching behaviours. Differences between raters were minimal and primarily reflected either the omission or over-identification of strategies, rather than fundamental disagreement on behaviour classification.

## 4 Discussion

This study aimed to develop OPTIn, an in-class observation instrument designed to measure physically active teaching strategies and overall teaching effectiveness. To the authors' knowledge, no existing tool specifically evaluates the implementation of physically active instructional methods, limiting our understanding of how such strategies impact student outcomes. The development of OPTIn followed a rigorous and systematic methodology, incorporating a comprehensive review of literature, expert input, iterative feedback, real-world piloting, and reliability and validity testing. These methodological steps were crucial for ensuring the tool's relevance, usability and practicality, thereby enhancing its effectiveness in accurately assessing the integration of physically active teaching methods.

The quality of teaching is a critical factor in influencing student outcomes, with specific instructional practices playing a central role in enhancing academic achievement and fostering learning progression (1, 5, 52). Various instruments have been developed to observe, assess, and provide feedback on teacher and instructional practices in the classroom (30, 53). These instruments assess classroom organisation, instructional support, and teacher competence (54), but largely reflect traditional teaching practices and fail to capture physically active instructional approaches, a gap OPTIn was designed to address.

OPTIn's observational assessments of practical teaching skills, inclusive of active strategies, enables evaluation, reflection and improvement in teacher quality. The integration of evidence-based teaching practices (5, 26, 51, 55, 56) within OPTIn strengthens its capacity to support meaningful improvements in teaching quality. By focusing observations on practices empirically linked to student achievement, OPTIn not only enhances the validity and reliability of evaluations but also provides targeted, actionable feedback for teachers. This approach supports ongoing professional development and promotes consistent, research-informed instructional improvement (24), ultimately contributing to better student learning, engagement and physical activity outcomes.

The multi-phase methodology employed in the development of OPTIn, ensured that the instrument was both grounded in existing research and responsive to expert input. In phase one and two, the expert reviewers provided critical feedback on the conceptual framework, clarity, and comprehensiveness of the instrument, ensuring it aligns with current knowledge and accurately measures the intended constructs (49). Pilot testing in phase three provided an early means of risk reduction, process optimisation, and project enhancement (57). The inclusion of inter- and intra-rater reliability testing, alongside content validity assessment against an expert criterion in phase four, strengthens the methodological rigour of the OPTIn instrument by demonstrating its consistency, accuracy, and appropriateness for capturing physically active teaching strategies in authentic classroom settings (49). This methodical approach enhanced its applicability for evaluating teaching practices, which is a key strength of this study.

Key findings provided by experts enabled an iterative process of instrument evolution, refined usability and ensured appropriateness for the target population (49). Expert recommendations included a self-reflection functionality, as well as an overall rating of teacher effectiveness. Self-assessment and reflective practice enhance teacher effectiveness by promoting ongoing professional growth, increasing self-awareness, and encouraging critical evaluation of teaching strategies (24). These practices enable teachers to identify strengths and areas for improvement, refine instructional methods, and adapt to diverse student needs, ultimately fostering more effective teaching and learning outcomes. Research shows that most teacher evaluation instruments are made available to the teacher before observation, used primarily as an evaluative tool, and rarely discussed in detail with the teacher following an observation (58, 59). In many cases, teachers report receiving their teaching evaluations via email with no additional contact from the evaluator, or report receiving no feedback at all (58). The option of using OPTIn for self-assessment via video, provides a powerful method for improving teaching. It enables immediate feedback, and encourages teachers to critically reflect on their practices, identify areas for growth, and make targeted adjustments that enhance instructional effectiveness and improves student outcomes.

The suggestion by experts to include an overall rating of teacher effectiveness was beneficial. Teacher effectiveness is the single greatest determinant in predicting how well students will do. Marzano (28) stated that most teacher evaluation systems do not adequately differentiate between effective and ineffective teachers, and these evaluation systems have not aided in teacher professional growth. OPTIn will enable the overall evaluation of teacher effectiveness, as well as identifying critical aspects of teaching that are predictive of student success.

While the methodology for developing the OPTIn instrument was rigorous, potential weaknesses included a limited and potentially biased expert pool, with only six researchers and nine experts providing feedback. The small sample size of pilot testers (five teacher educators) may not have fully captured the diversity of teaching contexts, and the reliance on expert opinions could overlook practical classroom challenges. Additionally, the methodology lacked longitudinal testing to assess the instrument's stability over time. Broadening the range of participants, including real-world teachers and considering diverse cultural and learning contexts and learners, could enhance the instrument's generalizability and applicability.

### 4.1 Practical implications

OPTIn has the potential to support educator professional learning by providing a structured, objective framework for observing, reflecting on, and improving the use of physically active teaching strategies alongside general teaching effectiveness. Its alignment with national teaching standards and evidence-based pedagogical practices makes it suitable for use in teacher education, professional development, and quality assurance contexts across primary, secondary, and tertiary education. By enabling consistent observation and targeted feedback, OPTIn may assist educators, teacher educators, and institutions to embed physically active learning more systematically within everyday teaching practice.

### 4.2 Future directions

The next steps are to undertake further testing with larger and more diverse samples across a range of educational settings to strengthen the robustness and generalisability of the instrument. Additional validation work is required to confirm its applicability across different teaching contexts, disciplines, and levels of teaching proficiency. Prior to widespread implementation, the instrument should be finalised through iterative refinement, informed by ongoing reliability and validity testing. Continued evaluation will be essential to ensure OPTIn remains responsive to evolving pedagogical practices and maintains its reliability and validity as a tool for evaluating and enhancing physically active teaching strategies in authentic classroom environments.

## 5 Conclusion

To the authors' knowledge, OPTIn represents the first instrument developed to use in-class observation to evaluate teaching practices, enabling specific and targeted feedback aimed at improving teaching quality and effectiveness, and ultimately enhancing the learning and health outcomes of students. The methodology used in development of the tool is both rigorous and systematic, incorporating expert guidance, iterative feedback, and pilot testing. The inclusion of education researchers and teacher educators ensured that the tool is firmly rooted in research and aligned with contemporary pedagogical standards. This approach guarantees that the final version of OPTIn provides a valuable means of measuring physically active teaching strategies and broader teaching effectiveness. Observation and objective evaluation are essential for strengthening teaching practices, as they facilitate reflective assessment and targeted improvements. As such, OPTIn may play a critical role in enhancing teaching effectiveness, ultimately contributing to the improvement of student learning outcomes.

## Data Availability

The raw data supporting the conclusions of this article will be made available by the authors, without undue reservation.
