# Supplementary material for: Observation of practical teaching skills instrument (OPTIn): transforming the way we observe, evaluate and improve physically active teaching strategies
Source: Front Sports Act Living. 2026 Feb 17;8:1748938. doi: 10.3389/fspor.2026.1748938 (PMC12954775; doi:10.3389/fspor.2026.1748938)
Supplement: Supplementary file 1 [file Datasheet1.pdf]

Observation of Practical Teaching Skills Instrument

OPT IN

TransformUs

Observation of Practical Teaching Skills Instrument

User Manual

IPAN Institute for Physical Activity and Nutrition

DEAKIN UNIVERSITY

TABLE OF CONTENTS

1 INTRODUCTION p. 1
Introducing OptIN and its benefits.

2 BACKGROUND p. 1
Exploring the need and purpose of OptIN.

3 INSTRUCTIONS p. 2
How to use OptIN.

4 CONSIDERATIONS p. 3
Ensuring consistency and purpose.

5 GLOSSARY p. 4
Key terms made simple.

6 CONTACT US p. 6
Contact the team.

7 REFERENCES p. 7
References used to develop the user manual.

1 INTRODUCTION

Welcome to the Observation of Practical Teaching Skills Instrument (OptIN).

The most important in-school factor driving student outcomes is teaching practice. OptIN is a comprehensive instrument for objectively evaluating practical teaching skills, and is suitable for observing all levels of teaching experience (e.g., undergraduate, graduate, proficient).

OptIN provides an objective measure of teaching practice that promotes classroom physical activity.

OptIN enables the simultaneous collection of data on:

- Practical teaching skill (e.g., pedagogical practices, classroom management).
- Evidence against the Australian Professional Standards for Teachers.
- Teacher competence and confidence.
- Student engagement.

Classroom physical activity can benefit students by:

- Improving the ability to stay on-task.
- Reducing disruptive behaviour.
- Improving motivation and engagement in learning.
- Improving academic performance.
- Improving wellbeing and health.

2 BACKGROUND

OptIN Enhances the Professional Learning Experience.

Prior to OptIN, there was no tool for making consistent, objective and informed real-time observations within the classroom. Research shows that professional learning that is 'job embedded' is 'often most beneficial' [1], with AITSL recommending that teachers 'spend more time on professional learning that is embedded into their daily practice' [1]. Furthermore, often professional learning that is conducted within the teachers teaching environment is thought to be most effective.

OptIN is intended to be used as a tool to record in-the-moment, objective and focused observations about teaching practice. It is intended to be used within the teachers own teaching environment, to provide on-the-spot feedback for teachers regarding their teaching practices. This feedback is intended to be used as a catalyst for reflection, professional conversation and professional development. OptIN is not a definitive assessment tool and does not provide rankings, outcomes, or a grading, and should not be used in this way.

3 INSTRUCTIONS

OptIN is a user-friendly tool for collecting focused observational data.

At the commencement of the class, you will be prompted to complete general information about yourself, the person you are observing, and the lesson you are observing.

OptIN will then prompt you to create an entry each time you observe active teaching strategy (eg., active break, active academic lesson).

After creating an entry of the observable active teaching strategy, you will then be prompted to record important contextual information about the teaching observation including:

- The phase of the lesson
- The instructional tool/s being used
- The teaching standard/s displayed
- The level of teaching competence and confidence, and
- The level of student engagement

Each observation will require a separate entry allowing for detailed evaluation. Observers (those creating the entries) should be mindful of language choices and should be cautioned about making judgements (rather than making observations).

Following the recording of each observation, select "Yes" to add another observation and continue the observation.

Following the final observation, select "No" to end the session.

Data will be collated to provide comprehensive evidence regarding the practical teaching skills of the teacher you have observed.

This data is intended to be used as a catalyst for reflection, professional conversation and professional development. OptIN can be used as data to support performance and development conversations or reflections and conversations about professional goals and professional development needs.

4 CONSIDERATIONS

OptIN is an Observational Tool.

In using OptIN within a school or organisation, users should prioritise consistency and purpose.

To ensure consistency between observations it is important that users adhere to a shared understanding of observation norms. Our considerations and suggestions to enhance consistency are as follows:

- **Observer position within the classroom:** Observers must have a clear view of the classroom, including teachers and students, and must be able to hear the teachers verbal instructions. In a traditional classroom where the teachers desk and white board is at the front of the classroom, and the student desks are set back from this space, it is recommended that the observer positions themselves within this setback space (space between the teachers desk/whiteboard and the students desks), on the opposite side to the teachers desk (or on one side of the classroom).
- **Conduct of the observer:** The observer should remain neutral in facial expression, gestures and mannerisms at all times. They should remain quiet and not involve themselves with any aspect of the lesson. The observers should be cautious of making targeted or purposeful eye contact with students or the teacher.
- **Length of observation:** OptIN is intended to be used for observations over a whole class period (period lengths can vary). This means that the observers should be present from the start to finish of the class.
- **Observations (what to look for):** Observers should familiarise themselves with the terms Active Breaks, Active Lessons and Active Environments (see Glossary and transformus.com.au) as well as the components, requirements and examples of each. Observers should look for moments during the class time where students are:
  - instructed/directed to be physically active
  - organised and structured to be physically active
  - purposefully physically active, and
  - where the physical activity is linked to the learning outcomes.

  If students are physically active (see glossary) in these ways, an observation entry should be made. Physical activity that is not directed, organised, structured, purposeful and linked to the learning outcomes (e.g., a student walking to the bin, a student going to the bathroom etc) does not require or constitute an observation entry.
- **Recording observations:** When creating observation entries, it is important that the observer uses neutral language, and only makes observations about what was seen, rather than inferences or judgements. Observations should not seek to rank or grade teacher skill or performance.

5 GLOSSARY

OptIN requires users to understand a number of key terms. Some of these terms have been hyperlinked for convenience.

Active Break: An Active Break is a short bout of physical activity used to enhance the overall learning experience. An Active Break is generally two to five-minutes in length (approximately). There are five types of Active Breaks - Energise, Manage, Transition, Structure and Learn (each types has been defined in the Glossary) [2].

Active Break - Energise: The purpose of an Energise Active Break is to alleviate the impact of long periods of sedentary learning. It is intended to increase blood flow to the brain and allow for students to become energised, refocused and re-engaged. Energise Active Breaks are usually characterised by moderate to higher intensity physical activity and may not be linked to the learning [2].

Active Break - Learn: The purpose of a Learn Active Break is to introduce, reinforce, consolidate, or demonstrate learning in a physically active way. A Learn Active Break embodies learning, and the movement/body becomes the learning (see Embodied Learning in the Glossary) [2].

Active Break - Manage: The purpose of a Manage Active Break is to mitigate the occurrences of 'off task behaviour', which result from a decline in focus, interest and concentration associated with lengthy periods of seated learning. Manage Active Breaks are usually characterised by low intensity physical activity, and they are implemented in a proactive manner - prior to 'off task behaviour' occurring [2].

Active Break - Structure: The purpose of a Structure Active Break is to allow students to have an active break, without disrupting the flow of the lesson or planned instructional practice. The use of a Structure Active Break is intended to increase physical activity by replacing or substituting a traditionally sedentary instructional practice, with an active version of the same instructional practice - physical activity is built into or integrated within the structure of the lesson [2].

Active Break - Transition: The purpose of a Transition Active Break is to allow intentional, structured and task orientated movement as students transition between one phase of the learning and the next, or one learning task and the next, or where students physically transition from one physical space to another [2].

5 GLOSSARY continued

Active Environments: An Active Environment is supportive of movement throughout the school day by shifting school attitude to normalise activity outside of traditional physical education and sport. Active Environments refer to the teaching environment (indoor and outdoor), as well as the broader school's environment (physical, cultural, policy etc). By creating active environments, the opportunities for active learning are enhanced and reinforced, and physical activity in many forms is promoted [3].

Active Lesson: An Active Lesson utilises incidental activity or embodied learning to change the delivery of a traditional, seated class lesson. Active Lessons involve a sustained period of physically active learning. This is where a particular phase of the lesson or learning task might be active. The period of physically active learning can vary, however, may be approximately 10 to 20 minutes in length [4].

APST: The APST or Australian Professional Standards for Teachers have been developed by the Australian Institute for Teaching and School Leadership (AITSL), and 'consist of seven standards, which teachers will meet at differing levels depending on their career stage and level of experience' [5]. 'The Australian Professional Standards for Teachers help you understand and develop your teaching practice and expertise across four career stages. By demonstrating the Standards you can have maximum impact on all learners' [6].

Embodied Learning: Learning through doing, where the body or movement itself becomes the learning tool [4].

Incidental Activity: Involves integrating movement into the structure of the lesson [4].

Observer: The person watching and recording the teaching and learning and recording observation entries.

Observation: A relevant and specific period of teaching and learning that is entered into OptIN.

Physical Activity (*physically active*): Any bodily movement produced by skeletal muscles that requires energy expenditure and refers to all movement including during leisure time, for transport to get to and from places, or as part of a person's work [7].

PST: A PST or Pre-Service Teacher is a someone who is studying to become a teacher (via a recognised university course or pathway), but who has not yet completed their teaching qualification and has not yet obtained full teaching registration.

6 CONTACT US

For all enquiries, feedback and support, contact the OptIN Team.

For research, function and use:

Associate Professor Natalie Lander

ARC DECRA Fellow Associate Professor
Institute for Physical Activity and Nutrition (IPAN)
School of Exercise and Nutrition Sciences
Deakin University

natalie.lander@deakin.edu.au

For educational support:

Jess Orr

TransformUs Education Lead
Institute for Physical Activity and Nutrition (IPAN)
School of Exercise and Nutrition Sciences
Deakin University

jess.orr@deakin.edu.au

For technical support:

Sam Lai

Project Manager (Associate Research Fellow)
TransformUs
Institute for Physical Activity and Nutrition (IPAN)
School of Exercise and Nutrition Science
Deakin University

s.lai@deakin.edu.au

Ned Weatherell

Research Assistant
TransformUs
Institute for Physical Activity and Nutrition (IPAN)
School of Exercise and Nutrition Science
Deakin University

ned.weatherell@deakin.edu.au
